# Design of High-Speed, Low-Power Sensing Circuits for Nano-Scale Embedded Memory

**DOI:** 10.3390/s24010016

**Published:** 2023-12-19

**Authors:** Sangheon Lee, Gwanwoo Park, Hanwool Jeong

**Affiliations:** 1 Department of Electronic Engineering, Kwangwoon University, Seoul 01897, Republic of Korea; leesang@kw.ac.kr (S.L.); gwanwoo0620@kw.ac.kr (G.P.); 2 Articron Inc., Ansan-si 15588, Republic of Korea

**Keywords:** static random-access memory, sensing circuit, offset voltage

## Abstract

This paper comparatively reviews sensing circuit designs for the most widely used embedded memory, static random-access memory (SRAM). Many sensing circuits for SRAM have been proposed to improve power efficiency and speed, because sensing operations in SRAM dominantly determine the overall speed and power consumption of the system-on-chip. This phenomenon is more pronounced in the nanoscale era, where SRAM bit-cells implemented near minimum-sized transistors are highly influenced by variation effects. Under this condition, for stable sensing, the control signal for accessing the selected bit-cell (word-line, WL) should be asserted for a long time, leading to increases in the power dissipation and delay at the same time. By innovating sensing circuits that can reduce the WL pulse width, the sensing power and speed can be efficiently improved, simultaneously. Throughout this paper, the strength and weakness of many SRAM sensing circuits are introduced in terms of various aspects—speed, area, power, etc.

## 1. Introduction

System-on-chip design encounters considerable challenges related to power consumption and latency, with an influence emanating from static random-access memory (SRAM) [1,2,3,4]. Thus, the efficient management of SRAM power consumption and the enhancement of SRAM access speed becomes highly important. Although reducing the supply voltage (V_DD_) proves effective in reducing power consumption, it introduces potential performance and stability trade-offs. In particular, the SRAM bit-cell, a circuit component for binary data storage, is typically constructed with near minimum-sized transistors to achieve high-density integration, resulting in significant performance variability due to process deviations [5,6,7,8]. Furthermore, to address read stability issues, read assist circuits are employed to suppress the word-line voltage, which can exacerbate performance degradation. Consequently, the optimization of SRAM circuits to minimize both power consumption and delay becomes crucial.

By analyzing the read operation, we can identify a method to simultaneously reduce power consumption and delay in SRAM. During the read operation, the bit-cell generates a voltage difference across the bit-line pair. Then, a sensing circuit measures this voltage difference and subsequently delivers the results to the external system. Importantly, the bit-line pair, which plays a fundamental role, has a significant capacitance, enough to make it the dominant contributor to both delay and power consumption during the read operation. Consequently, when a substantial voltage swing in the bit-line is necessitated for the read operation, it inevitably results in increased delays and power consumption. Thus, reducing the bit-line swing during the read operation can effectively decrease the power consumption and delay at the same time [9,10,11].

However, it is highly challenging to reduce bit-line voltage swing. This is because sensing circuits, especially the sense amplifier (SA) responsible for detecting bit-line swing, necessitate a sufficiently large bit-line voltage difference (Δ*V*_BL_) for precise operation. This need arises due to transistor mismatch within the SA, causing asymmetry in its characteristics. The minimum input voltage difference (in this case, Δ*V*_BL_) required for stable SA operation is known as the SA offset voltage (*V*_OS_). To reduce the Δ*V*_BL_, it becomes essential to lower the *V*_OS_.

Additionally, the SA is crucially utilized not only in SRAM but also in novel components, improving the efficiency of data processing [12,13,14,15,16,17,18,19,20,21]. SAs are used as row ADCs in [12,13,14], binary activation functions in [15,16,17], multilevel sense amplifiers in [18], four-bit flash ADCs in [19], and sensing circuits in [20,21]. Therefore, research on low *V*_OS_ for high accuracy, low power consumption, fast speed, and high integration for efficient performance is crucial for SAs.

Consequently, there are numerous prior research efforts proposed to reduce the *V*_OS_, the most important performance of SAs. The simplest method is to use larger width transistors for SAs, which can reduce the mismatch between paired transistors. However, this approach incurs area and power overhead. To reduce the *V*_OS_ while minimizing the area and power overhead, various offset reducing circuit techniques have been proposed [22,23,24,25,26,27,28,29,30,31,32,33,34,35,36,37,38,39,40,41,42,43,44,45,46,47]. This paper aims to conduct a comparative analysis of these circuits, explaining their effectiveness in reducing the *V*_OS_ and achieving power and performance benefits.

The rest of this paper is organized as follows: Section 2 provides essential background information on SRAM read operations and conventional SRAM sensing circuits, including an examination of their limitations. This foundation is crucial for understanding the subsequent content. Section 3 delves into comprehensive introductions of various previously researched SRAM sensing circuits designed to reduce the *V*_OS_, ultimately enhancing speed and power efficiency. Section 4 details a comparative analysis and discussion of the SRAM sensing circuits introduced in Section 3 from various perspectives.

## 2. Backgrounds on SRAM Read Operation and Conventional Sensing Circuits

**Figure 1:**
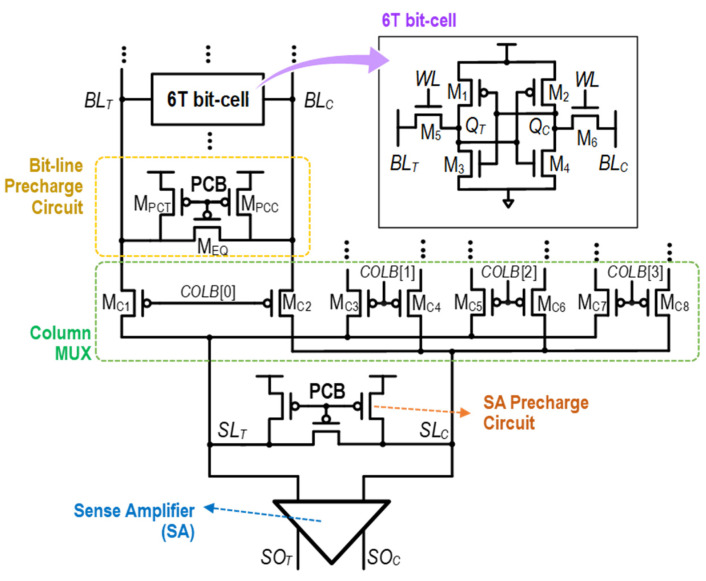
Simplified schematic of the conventional SRAM for the read operation.

Figure 1 presents the simplified circuits in the conventional SRAM for the read operation. In the following, we provide brief explanations for the structure and operation of each circuit shown in Figure 1.

At the top of Figure 1, the bit-cell is composed of six transistors. In this 6T bit-cell, two cross-coupled inverters are formed of M_1_, M_2_, M_3_, and M_4_ for storing and latching the binary data at two storage nodes, *Q*_T_ and *Q*_C_. The two access transistors, M_5_ and M_6_, serve as control elements that regulate connections between the bit-line pair (*BL*_T_ and *BL*_C_) and storage nodes (*Q*_T_ and *Q*_C_). When the *WL* activates (i.e., *WL* = 1), access transistors are turned on to connect bit-lines to storage nodes.

Next, the bit-line pre-charge circuit is shown, which is formed of *M*_PCT_, *M*_PCC,_ and *M*_EQ_. These transistors are controlled by the low-enable pre-charge trigger signal, *PCB*, with their gates connected. When *PCB* = 0, M_PCT_ and M_PCC_ are turned on to pre-charge *BL*_T_ and *BL*_C_ up to V_DD_, while *M*_EQ_ ensures that *BL*_T_ and *BL*_C_ are pre-charged to equal voltages.

The column multiplexer (MUX) implemented with M_C1_, M_C2_, …, M_C8_ selects one bit-line pair from multiple pairs (four pairs in Figure 1) and connects it to the SA input pair *SL*_T_ and *SL*_C_. The specific bit-line pair to be connected is determined by the column address signal, *COLB*[0:3], with only one of these signals set to low.

**Figure 2:**
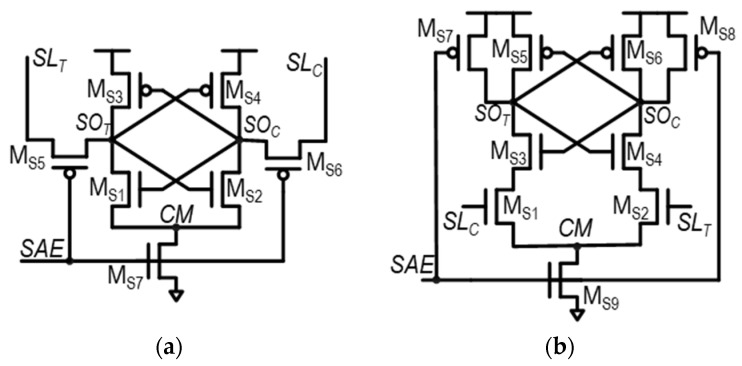
Schematic of two commonly used SAs in SRAM: (**a**) voltage-type latch SA (VLSA) and (**b**) current-type latch SA.

The SA plays a key role in the SRAM read operation. It amplifies the voltage difference between *SL*_T_ and *SL*_C_, converting it into a full-logic swing voltage. This amplified signal is then made available at the SA’s differential outputs—*SO*_T_ and *SO*_C_. Two commonly used conventional SA structures are the voltage-type latch SA (VLSA) and the current-type latch SA (CLSA), which are shown in Figure 2a,b, respectively [48]. Compared to VLSAs, CLSAs acquire SA input voltages, *SL*_C_ and *SL*_T_, through the gate of access transistors, M_S1_ and M_S2_. Therefore, the SA input voltage drives high impedance and less sensitivity to the timing mismatch. However, CLSAs have additional transistors for sensing operations. Therefore, CLSAs have lower speed performance, higher energy consumption, and a larger area, compared to VLSAs. The SA enable signal (SAE), connected to M_S5_–M_S7_ of VLSA and M_S7_–M_S9_ of CLSA, is utilized for triggering the amplifying operation of the SA.

**Figure 3:**
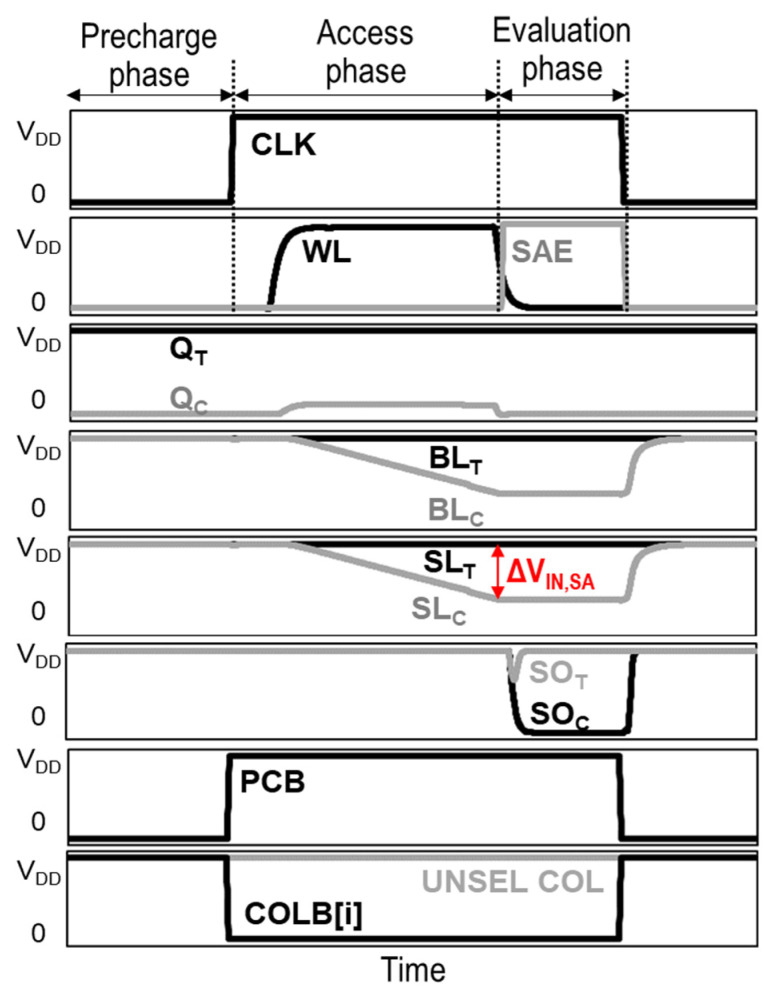
Operational waveforms for the read operation relevant signals in the conventional SRAM.

Figure 3 provides operational waveforms of relevant signals during the conventional SRAM read operation, divided into three phases: the pre-charge phase, the access phase, and the evaluation phase. In the pre-charge phase, the *PCB* becomes low, which pre-charges the bit-lines (*BL*_T_ and *BL*_C_) and SA inputs (*SL*_T_ and *SL*_C_) to V_DD_ through the bit-line pre-charge circuit and the SA input pre-charge circuit. Then, the access phase starts by making *PCB* = 1 to turn off the pre-charge circuits, while the *WL* for the selected bit-cell is asserted to reflect the data at Q_T_ and Q_C_ onto the bit-line pair of BL_T_ and BL_C_. Figure 3 shows an example of bit-cell storing datum “1” (Q_T_ = 1 and Q_C_ = 0). In this example, BL_T_ remains high while BL_C_ falls due to the bit-cell current through M_6_, creating a voltage difference between BL_T_ and BL_C_. By lowering the *COLB*[i] in the selected column, the column MUX transistors transfer only the selected bit-line pair voltage to the SA inputs, *SL_T_* and *SL_C_*.

**Figure 4:**
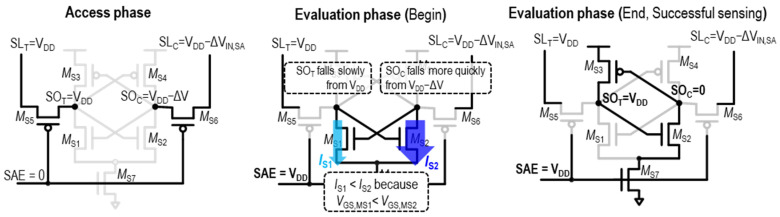
Description of VLSA operation for sensing datum “1”.

During the subsequent evaluation phase, the SA enable signal (*SAE*) becomes high to trigger the positive feedback configuration in the SA. In this manner, a small voltage difference between SL_T_ and SL_C_, ΔV_IN,SA_ (See Figure 3), is amplified into the digital voltage difference at SA output nodes *SO*_T_ and *SO*_C_. For example, the sensing operation of a VLSA in Figure 2a is shown in Figure 4.

When the sensing datum is “1”, the *SL*_T_ remains at *V*_DD_ while the *SL*_C_ decreases due to the bit-cell, reaching *V*_DD_ − ΔV_IN,SA_, as shown on the left side of Figure 4. The voltages at the SA outputs, *SO*_T_ and *SO*_C_, are equal to those at *SL*_T_ and *SL*_C_, respectively, through the pass transistors M_S5_ and M_S6_. During the subsequent evaluation phase, the *SAE* rises, and current flows through paired nFETs.

The FETs in the SA, M_S1_ and M_S2_, are depicted as *I*_S1_ and *I*_S2_ in the middle of Figure 4. At the beginning of the evaluation phase, the *V*_GS_ of M_S2_ (*SO*_T_ = *V*_DD_) is greater than that of M_S1_ (*SO_C_* = *V*_DD_ − ΔV_IN,SA_). Consequently, *I*_S2_ > *I*_S1_ makes *SO_C_* fall faster than *SO*_T_. This leads to positive feedback, formed by M_S1_–M_S2_–M_S3_–M_S4_. As a result, *SO*_T_ and *SO*_C_ eventually reach *V*_DD_ and 0 V, respectively, as shown on the right side of Figure 4, indicating a successful “1” datum sensing process.

**Figure 5:**
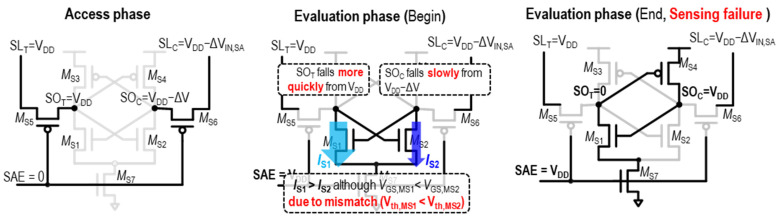
Description of sensing failure in VLSA for sensing datum “1”.

However, it is not always guaranteed that the SA operation is stably performed. In Figure 5, there is a scenario where sensing failure occurs. The access phase is the same as the previous normal sensing operation (the left side of Figure 5). However, when the evaluation starts by triggering the SA, as shown in the middle of Figure 5, problems can arise. It should be noted that, although the V_GS_ of M_S2_ (*SO*_T_ = V_DD_) is greater than the V_GS_ of M_S1_ (*SO_C_* = V_DD_ − ΔV_IN,SA_), *I*_S2_ < *I*_S1_. This can occur because there is a mismatch between the M_S1_–M_S2_ pair, specifically since the V_th_ of M_S1_ is lower than the V_th_ of M_S2_ [22]. Consequently, the *SO_T_* (initially V_DD_) falls more quickly than the *SO*_C_ (initially V_DD_ − ΔV_IN,SA_). Therefore, *SO*_T_ and *SO*_C_ end up with 0 V and *V*_DD_, respectively, meaning that sensing fails in attempting to sense datum “1”.

Here, the key point is that the mismatch between the paired transistors is responsible for the sensing failure. To prevent this sensing failure, ΔV_IN,SA_ should be large enough to compensate the effects of the transistor mismatch. This minimum required ΔV_IN,SA_ for stable sensing is the offset voltage in the SA, referred to as *V*_OS_, and necessitates that ΔV_IN,SA_ > *V*_OS_. This *V*_OS_ problem becomes severed in low-*V*_DD_ regions and is significantly affected by temperature [49,50]. To meet this condition, the *WL* pulse width is extended to achieve a sufficiently large ΔV_BL_, which, in turn, results in a large ΔV_IN,SA_. However, this increased ΔV_BL_ requirement not only causes delays but also raises power consumption, since more power is needed to pre-charge the significant capacitance of the BL pair, stemming from the combined effects of the long wire capacitance of the BL wire and the parasitic capacitance of the bit-cells.

Although employing large-sized transistors for sensing schemes can mitigate the mismatch problem, it incurs power, speed, and area overhead in the sensing stage [18]. In addition, the various replica bit-line delay or self-timed SAE generation techniques are proposed to minimize *WL* pulses [51,52,53,54,55,56,57,58], but their effects are limited because local variations cannot be considered. The speed and power issue due to the ΔV_BL_ requirement in SRAM becomes more severe in today’s advanced sub-nanometer technology nodes, because *WL*-suppressed assist circuits are widely used, which necessitates larger WL pulses for ΔV_BL_ requirements [59,60,61,62].

Therefore, it would be highly beneficial to reduce the *V*_OS_, as it would alleviate the demand for a large Δ*V*_BL_. In the following section, we describe SRAM sensing circuits designed to reduce the *V*_OS_ for the purpose of improving speed and power efficiency. We will explore these circuits in terms of their structure, operation, and key performance characteristics.

## 3. SRAM Sensing Circuits for Offset Reduction

### 3.1. Schmitt Trigger Sense Amplifiers

**Figure 6:**
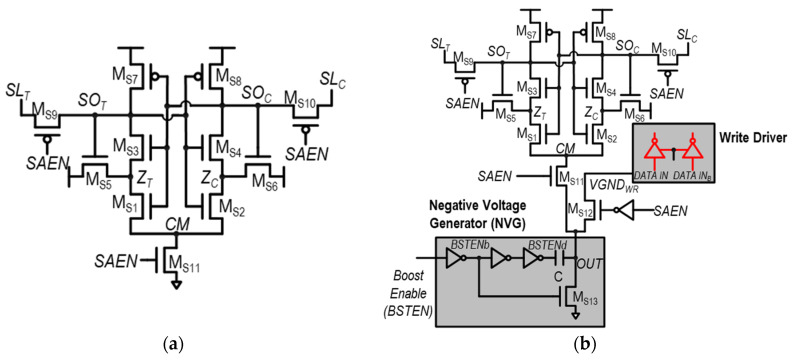
Schematics of (**a**) Schmitt trigger-based SA (STSA) and (**b**) the voltage-boosted STSA (VBSTSA).

Schmitt triggers are often used to improve the robustness of a standard inverter by modifying the switching threshold. Utilizing this feature, the authors in [24,25,26] proposed the Schmitt trigger-based SA (STSA) to reduce *V*_OS_, where one example structure is shown in Figure 6a. This structure intends to weaken the pull-down network of the inverter holding high voltages relative to that of the low-voltage inverter.

For example, when *SL*_T_ is *V*_DD_ while *SL*_C_ is V_DD_ − ΔV_IN,SA_ for datum “1” sensing, *SO*_T_ and *SO*_C_ become *V*_DD_ and *V*_DD_ − ΔV_IN,SA_, respectively, at the end of the access phase. When the evaluation phase starts with *SAE* rising, M_S5_ is more strongly turned on than M_S6_ because *SO*_T_ > *SO*_C_. Thus, the *Z_T_* node (the source of M_S3_) is more strongly pulled up than *Z_C_* (the source of M_S4_). In this manner, which adjusts not only the gate voltage but also controls the source voltages of M_S3_ and M_S4_ according to *SO*_T_ and *SO*_C_, the *V*_GS_ of M_S3_ is greatly suppressed. That is, the V_GS_ difference in two paired nFETs (M_S3_–M_S4_) in the STSA is larger than that in M_S1_–M_S2_ in the VLSA, which makes it more tolerant to the mismatch effects. In this manner, the STSA attempts to provide a reduced *V*_OS_ compared to the VLSA.

However, the STSA has a limited ability to reduce the *V*_OS_. This is because there are additional transistor pairs existing in the STSA; thus, the mismatch effect can be larger. In particular, the mismatch between M_S5_ and M_S6_ and the mismatch between M_S1_ and M_S2_, which are not present in the VLSA, increase the asymmetricity in the SA and increase the *V*_OS_. However, the circuit technique implemented in the STSA, performed by M_S1_, M_S2_, M_S5_, and M_S6_, effectively mitigates these mismatch effects, thereby compensating for the increase caused by the additional transistor pair. As a result, the final *V*_OS_ is reduced compared to the VLSA. Furthermore, the sensing delay is increased compared to the VLSA due to the use of a stacked nFET structure [26].

To mitigate the speed problem of STSAs, the voltage-boosted STSAs (VBSTSAs) are proposed [27], as shown in Figure 6b. In VBSTSAs, the negative voltage generator (NVG) used for the negative bit-line write-assist circuit is reutilized to accelerate the operation of STSAs. In the NVG, as the NVG operation starts, the BSTEN increases and the BSTENb decreases. Through the decreased BSTENb, M_S13_, which was holding OUT to V_SS_, is turned off, allowing OUT to reach a floating state. Subsequently, after M_S13_ is completely turned off, BSTENd, delayed through inverters, decreases and OUT is lowered to a negative voltage through a coupling capacitor, C. Note that BSTENd should decrease after the M_S13_ is fully turned off. Therefore, sufficient delay should be provided by the inverter in the NVG. Specifically, the ground voltage for the SA is pulled down to the negative voltage at the rising edge of the SAE, or 0 V otherwise. This is realized by making the switch, which is turned on only when the SAE is high, delivering the negative voltage generated by the NVG. Although sensing speed can be enhanced in this manner, it incurs a significant amount of power overhead. In addition, NVGs are not always used for write-assist circuits; other types of write-assist circuit, such as cell voltage collapse write assist, do not use NVGs.

### 3.2. Hybrid Latch-Type Sense Amplifiers

**Figure 7:**
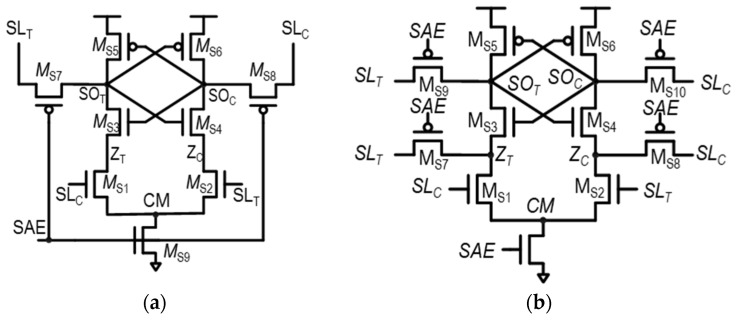
Schematics of two representative hybrid latch-type SAs: (**a**) variation-tolerant SA (VTSA) in [32] and (**b**) hybrid latch-type SA-QZ (HYSA-QZ) in [33].

Some previously proposed SAs combine the features of VLSAs and CLSAs to reduce the *V*_OS_, which can be referred to as hybrid latch-type SAs (HYSA) [28,29,30,31,32,33]. Figure 7a shows one example of an HYSA proposed in [32], the variation-tolerant SA (VTSA). For consistency in explanation with other structures, the polarity in this VTSA example is reversed from the original structure. The VTSA is primarily based on the CLSA structure but also incorporates features of a VLSA. Specifically, the SA outputs, *SO*_T_ and *SO*_C_, are pre-charged to the SA inputs, *SL*_T_ and *SL*_C_, using pass transistors M_S7_ and M_S8_.

When comparing VTSAs with VLSAs, a notable difference is observed in the pull-down networks of the positive feedback configurations in the SA. In the VTSA, these networks, consisting of M_S3_ and M_S4_, are not directly connected to the *CM* node as in the VLSA. Instead, they are connected to *Z_T_* and *Z_C_* nodes, as shown in Figure 7a. These nodes are pulled down by M_S1_ and M_S2_, respectively, with their gates controlled by *SL*_C_ and *SL*_T_. This configuration effectively adjusts the *V_GS_* of M_S3_ and M_S4_ for proper sensing.

The detailed operation of the VTSA is as follows: During the access phase, when *SAE* = 0 and datum “1” is being sensed, the *SL_T_* is at V_DD_, and *SL*_C_ is at V_DD_ − ΔV_IN,SA_, making *SO*_T_ and *SO*_C_ pre-charged to V_DD_ and V_DD_ − ΔV_IN,SA_, respectively, through M_S7_ and M_S8_, similar to the VLSA. Additionally, the gate voltages of M_S1_ and M_S2_, V_G,MS1_ and V_G,MS2_, become V_DD_ − ΔV_IN,SA_ and V_DD_, respectively. When the evaluation phase begins with *SAE* = 1, *Z*_T_ and *Z*_C_ are pulled down by M_S1_ and M_S2_, respectively. In this configuration, since *SL*_T_ > *SL*_C_, M_S1_ can drive more current than M_S2_, resulting in *Z_C_* being pulled down more strongly than *Z_T_* (i.e., *Z*_T_ > *Z*_C_). As a result, compared to the VLSA, the difference between *V_GS,MS3_* and *V_GS,MS4_* is lager in the VTSA, indicating that the amplification can be more stabilized, and thus, *V*_OS_ can be reduced. This is due to adjustments made not only in the gate voltage conditions of M_S3_ and M_S4_ (V_G,MS3_ < V_G,MS4_), but also in their source voltage conditions (V_S,MS3_ > V_S,MS4_).

However, the VTSA has an additional pair of nFET transistors compared to the VLSA—M_S1_ and M_S2_—involved in the initial amplification of signals. This additional pair not only incurs area overhead but also potentially increases the mismatch effects. That is, the mismatch between M_S1_ and M_S2_, which does not need to be considered in VLSAs, can result in unintentional changes in *Z_T_* and *Z_C_* and degrade the sensing stability. In addition, stacked nFETs degrade the sensing delay and power consumption, like STSAs.

Figure 7b shows another example of an HYSA, the HYSA-QZ, which is proposed in [33]. This structure more aggressively pre-charges the internal nodes of the SA than the VTSA. The notation of QZ here means that not only output nodes (Q), but the internal nodes between the M_S1_–M_S2_ pair and M_S3_–M_S4_ pair (Z) are also pre-charged to SA inputs in a direction for precise sensing. As shown in Figure 7b, not only *SO_T_* and *SO_C_* are pre-charged to *SL_T_* and *SL_C_*, but also *Z_T_* and *Z_C_* are pre-charged to *SL_T_* and *SL_C_*, respectively. In this manner, the bias condition of the SA becomes more favorable for accurate sensing than the VTSA.

### 3.3. Capacitor-Based Offset-Compensated SAs

**Figure 8:**
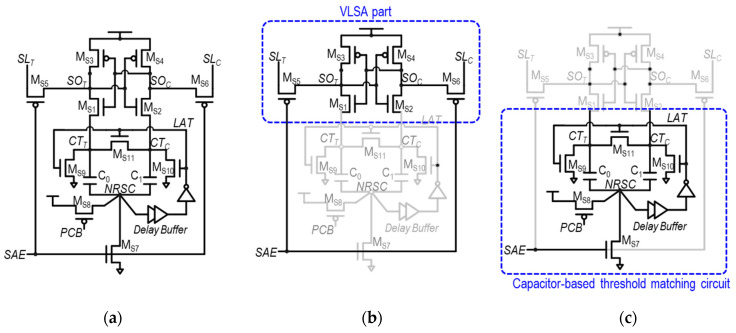
(**a**) Schematic of capacitor-based threshold-matching SA (TMSA), (**b**) VLSA part in TMSA, and (**c**) capacitor-based threshold-matching circuit part.

Several previously proposed SAs have addressed transistor mismatches by employing capacitors [34,35,36,37,38,39,40]. These capacitors capture the mismatches between paired transistors, and the stored mismatch information is subsequently utilized to bias the internal nodes of the SA for compensation. Figure 8a illustrates the configuration of a capacitor-based threshold-matching SA (TMSA), as presented in [38].

As demonstrated in Figure 8b,c, the TMSA comprises two main components: a VLSA part and the capacitor-based threshold-matching part. The primary goal of the TMSA is to compensate the mismatch between the M_S1_–M_S2_ pair, which is the most critical pair in a VLSA. This correction is accomplished by initially sampling the *V*_th_ of M_S1_ and M_S2_—*V*_th,MS1_ and *V*_th,MS2_—during the pre-charge phase. Then, the sampled *V*_th,MS1_ and *V*_th,MS2_ are stored at the source nodes of M_S1_ and M_S2_. This ensures that the current through M_S1_ and M_S2_ during the amplification operation—I_S1_ and I_S2_—are independent to their V_th_ mismatch.

**Figure 9:**
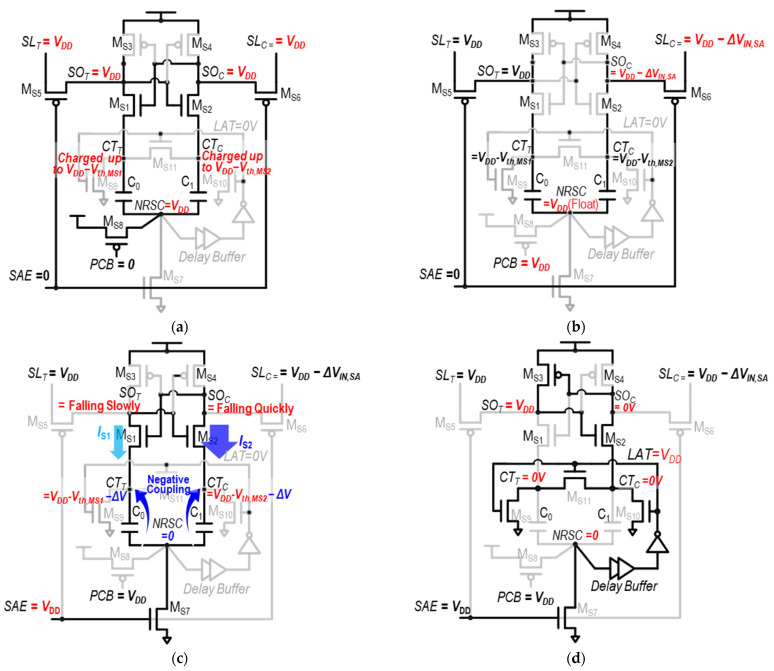
Four-step operation of TMSA: (**a**) pre-charge phase, (**b**) access phase, (**c**) evaluation phase, and (**d**) latching phase.

The detailed operation that achieves this objective is illustrated in Figure 9a–d, in the example of sensing datum “1”, with a comprehensive explanation provided as follows.

(1) Pre-charge phase (Figure 9a): During this phase, the input and output nodes of the SA—*SL*_T_, *SL*_C_, *SO*_T_, and *SO*_C_—are pre-charged to *V*_DD_. Then, the top-plate nodes of C_0_ and C_1_—*CT*_T_ and *CT*_C_—are pre-charged to *V*_DD_ − *V*_th,MS1_ and *V*_DD_ − *V*_th,MS2_, respectively, and M_S1_ and M_S2_ become turned off. This pre-charge is conducted under the assumption that *CT*_T_ and *CT*_C_ are initially at 0 V before pre-charging (the rationale for this will be explained). In addition, the common bottom-plate node for C_0_ and C_1_, *NRSC*, is pre-charged to *V*_DD_ by M_S8_, which is turned on by *PCB* = 0.
(2) Access phase (Figure 9b): In this phase, *SL_C_* is lowered and becomes *V*_DD_ − Δ*V*_IN,SA_ by the bit-cell, causing the *SO_C_* to also be *V*_DD_ − Δ*V*_IN,SA_. In addition, the *PCB* becomes high, so the common bottom-plate node of C_0_ and C_1_, *NRSC*, becomes float-high.
(3) Evaluation phase (Figure 9c): This phase starts with the *SAE* rising, turning on M_S7_, so the *NRSC* is pulled down. This results in negative capacitive voltage couplings from *NRSC* to *CT_T_* and *CT_C_*, through C_0_ and C_1_, respectively. Thus, *CT_T_* and *CT_C_* are decreased by Δ*V*, meaning that *CT*_T_ and *CT*_C_ are changed into *V*_DD_ − *V*_th,MS1_ − Δ*V* and *V*_DD_ − *V*_th,MS2_ − Δ*V*, respectively. These turn on M_S1_ and M_S2_, where the overdrive voltage (*V*_OV_ = *V*_GS_ − *V*_th_) of M_S1_ and M_S2_—*V*_OV,MS1_ and *V*_OV,MS2_—become as follows:
  *V*_OV,MS1_ = *V*_GS,M1_ − *V*_th,M1_ = *V*(*SO*_C_) − *V*(*CT*_T_) − *V*_th,MS1_
  = (*V*_DD_ − Δ*V*_IN,SA_) − (*V*_DD_ − *V*_th,MS1_ − Δ*V*) − *V*_th,MS1_ = Δ*V* − Δ*V*_IN,SA_
  *V*_OV,MS2_ = *V*_GS,M2_ − *V*_th,M2_ = *V*(*SO*_T_) − *V*(*CT*_C_) − *V*_th,MS2_
  = *V*_DD_ − (*V*_DD_ − *V*_th,MS2_ − Δ*V*) − *V*_th,MS2_ = Δ*V*
  The noticeable point is that *V*_OV,MS1_ and *V*_OV,MS2_, which determine *I_S_*_1_ and *I_S_*_2_, are independent of V_th,MS1_ and V_th,MS2_, respectively. Thus, even in the presence of a mismatch between V_th,MS1_ and V_th,MS2_, *I*_S1_ and *I*_S2_ can be stably generated (e.g., *I*_S1_ < *I*_S2_ for datum “1“ sensing as in Figure 9c) at the beginning of the evaluation phase. This renders the TMSA to be notably more robust than the conventional VLSA, leading to a reduced *V*_OS_.
(4) Latching phase (Figure 9d): After the *NRSC* becomes low in the evaluation phase, this change in *NRSC* propagates to make *LAT* = V_DD_ through a delay buffer, which starts the latching phase. In this phase, *CT*_T_ and *CT*_C_ become 0 V, so *SO*_T_ and *SO*_C_ can latch the sensing results at the full digital level. This state is kept until the next pre-charge phase. Here, one can see that *CT*_T_ and *CT*_C_ are 0 V, and they are to be charged up to *V_DD_* − *V_th,MS_*_1_ and *V_DD_* − *V_th,MS_*_2_, respectively, in the next pre-charge phase.

Although the TMSA effectively reduces the *V*_OS_ by compensating the mismatch between M_S1_ and M_S2_, there are several shortcomings in this structure. First, the structure is still under the effect of a mismatch between capacitors, C_0_ and C_1_. The mismatch, however, is typically much smaller than the transistor V_th_ mismatch. Second, the implementation of capacitors and delay buffers in the TMSA results in a significant increase in power consumption and area requirements. In particular, a sufficiently large Δ*V* is necessary to turn on M_S1_ and M_S2_ in the early stage of the amplification stage; it is inevitable to employ large capacitors for C_0_ and C_1_. However, by placing the metal–oxide–metal (MOM) capacitors on top of the circuit layout, the area overhead can be avoided [39]. Consequently, a significant amount of power is required to charge up the *NRSC* from 0 V to *V*_DD_ in the pre-charge phase.

**Figure 10:**
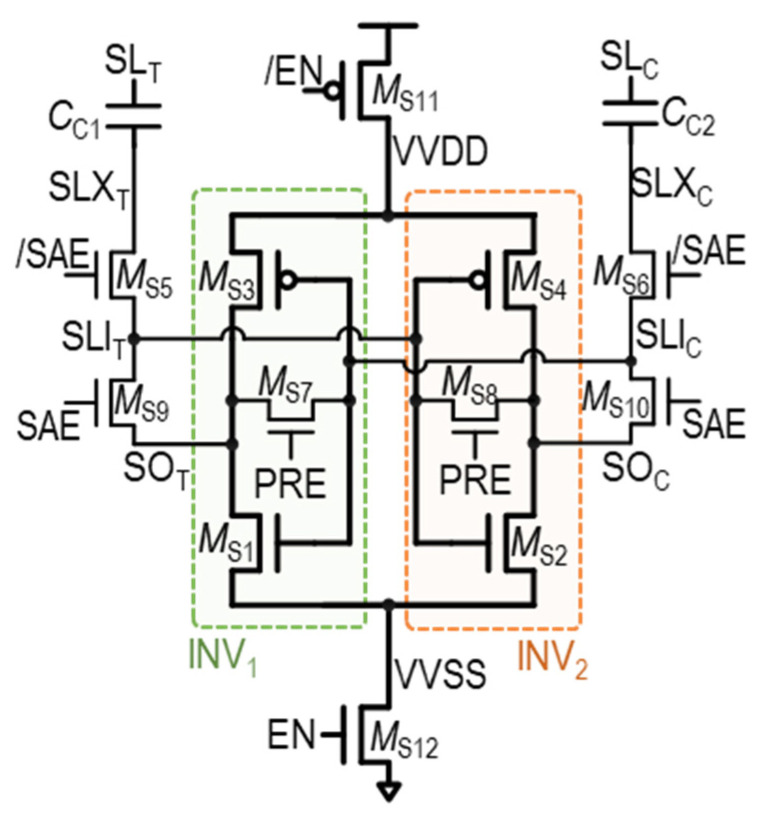
Structure of VTS-SA.

As an alternative approach, the variation-tolerant small-signal SA (VTS-SA) is proposed in [39], specifically addressing mismatches between the two inverters in the SA. This is achieved through the utilization of capacitors at the input acceptance part. The structure of the VTS-SA is shown in Figure 10 below.

The VTS-SA is based on a VLSA composed of M_S1_–M_S2_–M_S3_–M_S4_, while the SA input nodes, *SL*_T_ and *SL*_C_, are accepted through coupling capacitors C_C1_ and C_C2_, respectively. By utilizing capacitors, the VTS-SA can capture and store the trip points of two inverters in SA-INV_1_ (M_S1_ and M_S3_) and INV_2_ (M_S2_ and M_S4_), shown in Figure 10. By biasing the two inverters with their respective trip points, the two inverters become highly sensitive to small voltage input variations. That is, even small input voltage changes can push the inverters to switch their output states. This enhanced voltage gain of the inverters contributes to the improved speed of the SA. Furthermore, trip-point biasing in the VTS-SA serves another crucial purpose: it allows the SA to adapt and account for process variations within the inverters. By individually setting the trip points, the VTS-SA makes each inverter operate primarily in response to input changes, minimizing its dependence on process variations as much as possible.

**Figure 11:**
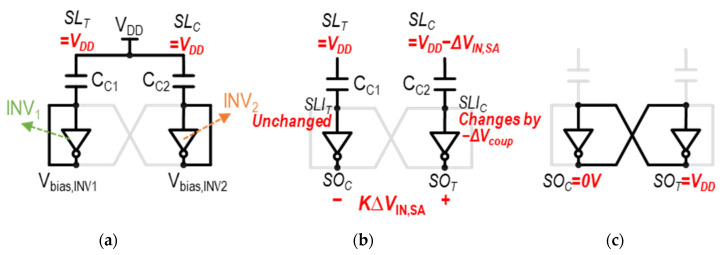
Three operation phases of VTS-SA: (**a**) trip-point bias, (**b**) access phase, and (**c**) evaluation phase.

The detailed operations of the VTS-SA are illustrated in Figure 11a–c, where there are three main operation phases: (1) the trip-point bias phase, (2) the access phase, and (3) the evaluation phase.

(1) Trip-point bias phase (Figure 11a): In this phase, the input and output are shorted in INV_1_ and INV_2_ of the SA. As a result, the input and output of INV_1_ and INV_2_ are set to their respective trip points—V_bias,INV1_ and V_bias,INV2_. This is accomplished by turning on the *M*_S7_ and *M*_S8_ transistors through *PRE* = 1, while also turning on the header and footer switches *M*_S11_ and *M*_S12_ with *EN* = 1. In addition, *SAE* = 0 in this phase, to make the bottom plate of the coupling capacitors, *SLI_T_* and *SLI_C_*, also be equal to the trip points of the inverters.
(2) Access phase (Figure 11b): In this phase, the input–output connections are disconnected, and the two trip-point-biased inverters are ready to accept changes in *SL*_T_ and *SL*_C_ through capacitive couplings. Specifically, when sensing datum “1”, as demonstrated in Figure 11b, *SL*_C_ is decreased by Δ*V*_IN,SA_. Then, *SLI*_C_ is decreased by Δ*V*_coup_ through capacitive coupling via C_C1_. Due to trip-point bias, this input change of INV2 leads to a significant change in the output of INV_2_, *SO_T_*. As a result, an amplified voltage difference is observed between *SO*_T_ and *SO*_C_, which is K × Δ*V*_IN,SA_, where K > 1. It is important to note that, as previously mentioned, because the inverters are biased to their respective trip point, the output change is almost only determined by the input change, while largely independent to the process variations.
(3) Evaluation phase (Figure 11c): In this phase, the *SAE* becomes high; thus, the two inverters are connected in a cross-coupled fashion, by turning on M_S10_ and M_S9_. At the same time, the two cross-coupled inverters are isolated from the input by turning off M_S5_ and M_S6_. Through the positive feedback of the cross-coupled inverters, the final data are latched onto *SO*_T_ and *SO*_C_ at the full digital level, similar to the operation of other SAs.

Although the VTS-SA tries to reduce the *V*_OS_ by capturing the mismatch between INV_1_ and INV_2_ through trip-point biasing, there are several limitations to this structure. First, the mismatch between M_S5_–M_S6_, M_S7_–M_S8_, and M_S9_–M_S10_ are newly introduced in this structure, which limits *V*_OS_ reduction. Second, similar to the TMSA, the VTS-SA is still affected by mismatches between C_C1_ and C_C2_, although it is less influential than the transistor mismatch. Third, because the input voltage should be transferred through capacitive coupling, not all of the Δ*V*_IN,SA_ is delivered to the SA. This inefficiency contributes to an increase in effective *V*_OS_. Fourth, the trip-point biasing process should be completed before the Δ*V*_IN,SA_ appears between *SL*_T_ and *SL*_C_. This requirement potentially increases the circuit complexity. In addition, the short current from V_DD_ to V_SS_ is inevitable during the trip-point biasing, resulting in high power consumption.

**Figure 12:**
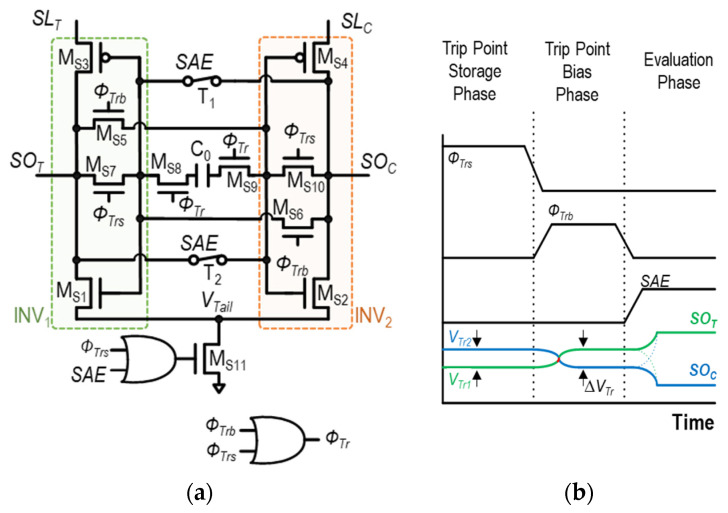
(**a**) Schematic of CSA_COC_ and (**b**) operation waveforms of three control clock signals.

The current-mode SA with a capacitive offset correction (CSA_COC_) structure proposed in [40] utilizes a single capacitor for storing the trip points of inverters, so it is free from capacitor mismatch effects. The schematic of the CSA_COC_ is shown in Figure 12a, and the operation waveforms of its three main control clock signals—the trip-point storage enable, *Φ*_Trs_; the trip-point bias enable, *Φ*_Trb_; and the sense enable, *SAE*—are illustrated in Figure 12b.

**Figure 13:**
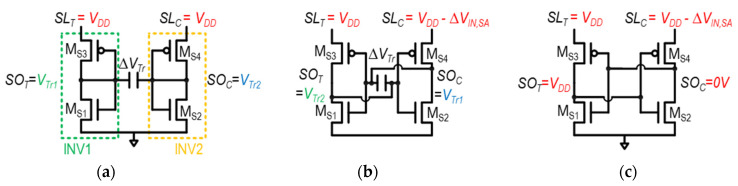
Three operation phases of CSA_COC_: (**a**) trip-point storage phase (*Φ*_Trs_ = 1), (**b**) trip-point bias phase (*Φ*_Trb_ = 1), and (**c**) evaluation phase (*SAE* = 1).

The key concept of the CSA_COC_ is to store the difference in the trip point voltages of the two inverters, INV_1_ and INV_2_, in Figure 12a. The difference in the trip point voltages of the two inverters, Δ*V*_Tr_ = *V*_Tr1_–*V*_Tr2_, is stored across the single capacitor, C_0_. Then, the two inverters are biased to compensate the trip-point difference, effectively correcting for the mismatch. The operation of CSA_COC_ unfolds in three phases, as illustrated in Figure 13a–c, with explanations for each provided as follows.

(1) Trip-point storage phase (*Φ_Trs_* = 1, Figure 13a): In this phase, *SL*_T_ and *SL*_C_ are pre-charged to V_DD_, and the input and output of each inverter, INV_1_ and INV_2_, are shorted. In this manner, the trip points of INV_1_ and INV_2_, V_Tr1_ and V_Tr2_, are captured and stored at the input and output nodes of the respective inverters, as shown in Figure 13a. It is accomplished by turning on M_S7_, M_S8_, M_S9_, and M_S10_ while turning off T_1_, T_2_, M_S5_, and M_S6_. The difference between two inverter trip points, Δ*V*_Tr_, is stored across the capacitor, C_0_.
(2) Trip-point bias phase (*Φ*_Trb_ =1, Figure 13b): During this phase, the input and output of INV1 and INV2 are disconnected by turning off M_S7_ and MS_10_. Subsequently, by utilizing the Δ*V*_Tr_ stored in C_0_ in the previous phase, the input of each inverter is held as its respective trip point, while INV_1_ and INV_2_ are configured in the cross-coupled connection. For example, the input of INV_1_ is kept as *V*_Tr1_, while it is connected to the output of INV_2_ (=*SO*_c_), and vice versa. This is achieved by turning on M_S5_ and M_S7_ while turning off MS_11_. Then, the voltage difference is made between *SL*_T_ and *SL*_C_ by the bit-cell, and develops the differential current through M_S3_ and M_S4_.
(3) Evaluation phase (*SAE* = 1, Figure 13c): In this phase, the two cross-coupled inverters are disconnected from C0 by turning off M_S8_ and M_S9_. Simultaneously, the positive feedback of the cross-coupled inverters is initiated by turning on M_S11_, T_1_, and T_2_. As a result, the full digital voltage level appears at two differential outputs of the SA, *SO*_T_ and *SO*_C_.

The CSA_COC_ is immune to capacitor mismatch due to use of a single capacitor, unlike the TMSA and VTS-SA. However, compared to the previous SAs in which the voltage between *SL*_T_ and *SL*_C_ is transferred to *SO*_T_ and *SO*_C_ through fully turned-on pFETs during the access phase, in the CSA_COC_, the voltage difference between *SO*_T_ and SOC follows that of *SL*_T_ and *SO*_C_ through partially turned-on pFETs (current-based). This leads to voltage loss, effectively increasing the *V*_OS_. In addition, there are numerous required switches and a control signal generation logic, which increases the circuit design complexity with power and area overhead.

### 3.4. Offset-Compensated Pre-Amplifiers

**Figure 14:**
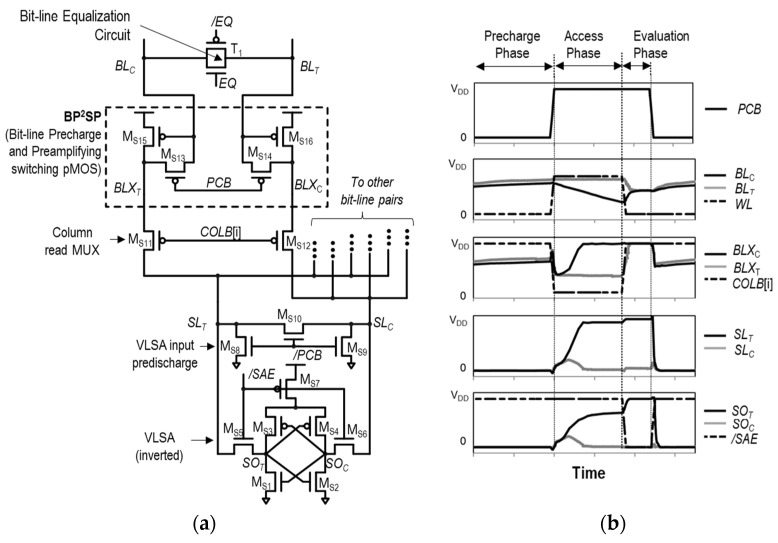
(**a**) Schematic of BP^2^SP and (**b**) its operational waveforms.

Another approach in offset compensation is the use of pre-amplifiers that amplify the bit-line signal preceding the SA stage, as seen in [41,42,43,44]. Instead of directly modifying the SA structure, these additional offset-compensating pre-amplifiers are employed in front of the SA. This allows for the required offset compensation while maintaining the original SA structure. One such example is the bit-line pre-charge and pre-amplifying switching pFET circuit (BP^2^SP), with its structure and key operational waveforms depicted in Figure 14a,b.

As shown in Figure 14b, BP^2^SP is operated in three phases, as explained below.

(1) Pre-charge phase (*PCB* = 0): In this phase, M_S13_ and M_S14_ in BP^2^SP are turned on to pre-charge *BL_C_* and *BL_T_*, respectively. This pre-charges *BL*_C_ and *BL*_T_ to *V*_DD_ − *V*_th,MS15_ and *V*_DD_ − *V*_th,MS16_, respectively, through a diode connection. It ensures that M_S15_ and M_S16_ have *V*_GS_ = *V*_th_, allowing them to turn on immediately, regardless of *V*_th_ variations, when *BL_C_* or *BL_T_* is discharged in the subsequent phase. This compensates the V_th_ mismatch between M_S15_ and M_S16_. In the SA side, *SL*_T_ and *SL*_C_ are pre-discharged to 0 V through M_S8_ and M_S9_.
(2) Access phase (*PCB* = 1, *WL* = 1): During this phase, the data stored in the selected bit-line are reflected to the *BL*_T_ and *BL*_C_. In the example shown in Figure 14b, datum “1” is sensed, so the *BL*_T_ remains close to its pre-charge level, *V*_DD_ − *V*_th,MS16_, while *BL*_C_ decreases from *V*_DD_ − *V*_th,MS15_. Because the *BL*_C_ is pre-charged at V_DD_ − V_th,MS15_, M_S15_ turns on instantly as soon as the *BL*_C_ decreases. This causes the *BLX*_T_ to increase rapidly. Simultaneously, the *COLB* is lowered to enable the column MUX, resulting in *SL*_T_ increasing and *SL*_C_ remaining at 0 V. As shown in Figure 14b, this phase effectively pre-amplifies the voltage difference between *BL*_T_ and *BL*_C_ to the voltage difference between *SL*_T_ and *SL*_C_.
(3) Evaluation phase (SAE = 1): In this phase, the *SAE* is raised, meaning /*SAE* is lowered. Consequently, the VLSA is enabled to store the final sensing data in the form of a full digital voltage at the *SO*_T_ and *SO*_C_ nodes. In addition, during this phase, the bit-line equalization circuit—transmission gate T_1_—is activated to equalize BL_T_ and BL_C_. This ensures that the subsequent pre-charge operation of BL_T_ and BL_C_ can start with both bit-lines having the same low voltage level as the initial condition. This equalization step is important for maintaining consistency in the subsequent memory operation.

The operation principle of BP^2^SP is to use the same pFETs for using pre-charge bit-line and pre-amplify bit-line voltages. Specifically, by pre-charging the bit-line to capture the *V*_th_ variation of the pre-amplifying pFETs, these pre-amplifying pFETs can instantly turn on in response to bit-line pair voltage development. This allows the amplified voltage to be observed at SL_T_ and SL_C_, reducing the required ΔV_BL_ for stable sensing, leading to improvements in speed and power efficiency. However, to make bit-line pairs to *V*_DD_ − *V*_th_, it is necessary to ensure that the bit-line voltages are sufficiently lower than *V*_DD_ − *V*_th_ before pre-charge. This requirement increases the circuit complexity, especially when the memory is awakened from power-down mode or standby mode. In addition, after pre-charging the bit-line pair to V_DD_ − V_th_, the bit-lines become floating, making them susceptible to noise. Moreover, the initial V_GS_ condition of pre-amplifier pFETs can significantly vary according to the pre-charge period, which means that the overall speed of the read operation is highly affected by the pre-charge time.

**Figure 15:**
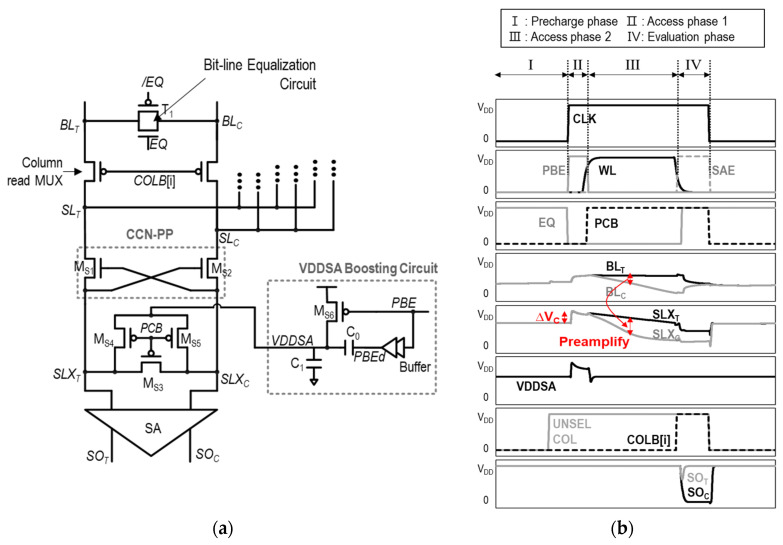
(**a**) Schematic of CCN-PP and (**b**) its operational waveforms.

In [43], another pre-amplifier circuit for SRAM, the cross-coupled nFET pre-amplifier and pre-charge circuit (CCN-PP), is presented. The structure and operational waveforms of the CCN-PP are shown in Figure 15a,b. As depicted in Figure 15b, the CCN-PP operates in four phases.

(1) Pre-charge phase (*PBE* = 0, *PCB* = 0): During this phase, the pre-charging boost enable signal (PBE) and PCB are low, so the SA input pre-charge circuit (M_S3_–M_S4_–M_S5_) and M_S6_ are turned on. This maintains *VDDSA* as *V*_DD_, while SLX_T_ and SLX_C_ are pre-charged to *V*_DD_. It should be noted that, unlike the conventional pre-charge operation, all the column MUX transistors and bit-line equalization circuits (T_1_) are turned on. As a result, SL_T_, SL_X_, BL_T_, and BL_C_ are pre-charged through the CCN-PP. Because the CCN-PP is composed of nFETS, there a threshold voltage drop for pre-charging voltages. That is, *BL*_T_ and *BL*_C_ are pre-charged to *V*_DD_ − min(*V*_th,MS1_, *V*_th,MS2_).
(2) Access phase 1 (*PBE* = 1): During this phase, the unselected column MUX transistors are turned off and the *PBE* is raised. As a result, M_S6_ is turned off and then the PBEd rises, boosting the VDDSA into V_DD_ + ΔV_C_ through C_0_ coupling. Thus, the SA inputs, *SLX*_T_ and *SLX*_C_, are also pre-charged to V_DD_ + ΔV_C_. Accordingly, BL_T_ and BL_C_ can be slightly raised. In this phase, the WL is activated, so BL_T_ and BL_C_ start to be developed according to bit-cell data.
(3) Access phase 2 (PBE = 0, PCB = 1): With PCB rising, SLX_T_ and SLX_C_ are affected by the change in BL_T_ and BL_C_ through the CCN-PP. For example, when accessing the datum “1”, as shown in Figure 15b, BL_C_ and SL_C_ decrease, leading M_S2_ to be turned on while M_S1_ is kept turned off. The turned-on M_S2_ makes SLX_C_ fall while SLX_T_ is kept high, close to V_DD_ + ΔV_C_. Due to the positive feedback nature of cross-coupled nFETs, the voltage difference between SLX_T_ and SLX_C_ is larger than that of BL_T_ and BL_C_, meaning that the bit-line voltage is pre-amplified.
(4) Evaluation phase (SAE = 1): High SAEs activate the SA to latch the data at SA outputs, *SO*_T_ and *SO*_C_. In addition, similar to BP^2^SP, the bit-line equalization circuit is activated to provide proper bit-line initial conditions for the subsequent pre-charge phase.

Unlike BP^2^SP, the initial V_GS_ of pre-amplifier transistors in the CCN-PP are determined by access phase 1. Thus, the performance is less dependent on the pre-charge period, so a stable speed can be provided with the CCN-PP. However, as in BP^2^SP, the CCN-PP still suffers from floating BL_T_ and BL_C_ during the pre-charge phase. In addition, the CCN-PP cannot compensate the mismatch between M_S1_ and M_S2_, which is an inferior point compared to BP^2^SP. In addition, utilizing the VDDSA boosting circuit can incur a significant amount of power and area overhead.

**Figure 16:**
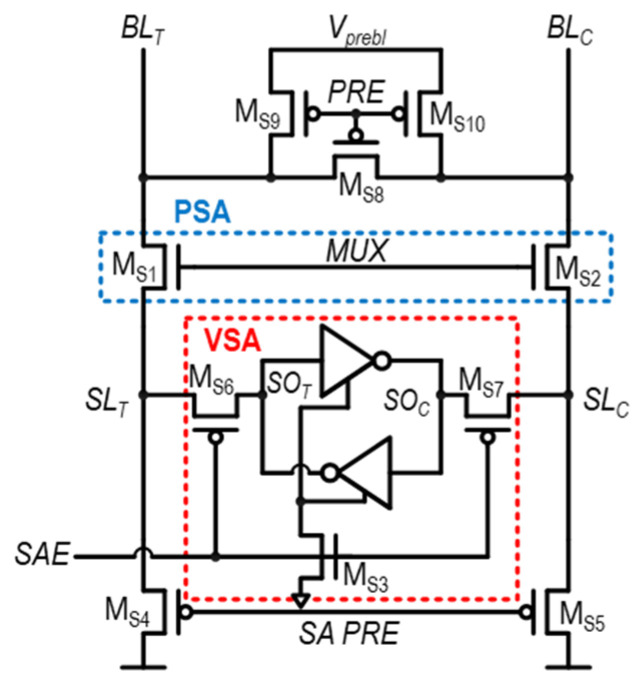
Structure of OCCSA.

In [44], the offset-cancelled current SA (OCCSA) is proposed. As shown in Figure 16, the OCCSA uses nFET MUX transistors instead of pFET MUX transistors. Here, the nFET MUX (PSA) operates as a common-gate amplifier, so it effectively pre-amplifies the BL. To bias these PSAs properly with offset-compensating features, BL_T_ and BL_C_, the BL should be pre-charged lower than V_DD_ − V_th,MS1_ and V_DD_ − V_th,MS2_, respectively. To realize this, a separate supply voltage, V_prebl_, is required. However, the incorporation of this new voltage source is highly costly due to its substantial power and area overheads, making the circuit impractical for actual implementation.

### 3.5. Other Structures

**Figure 17:**
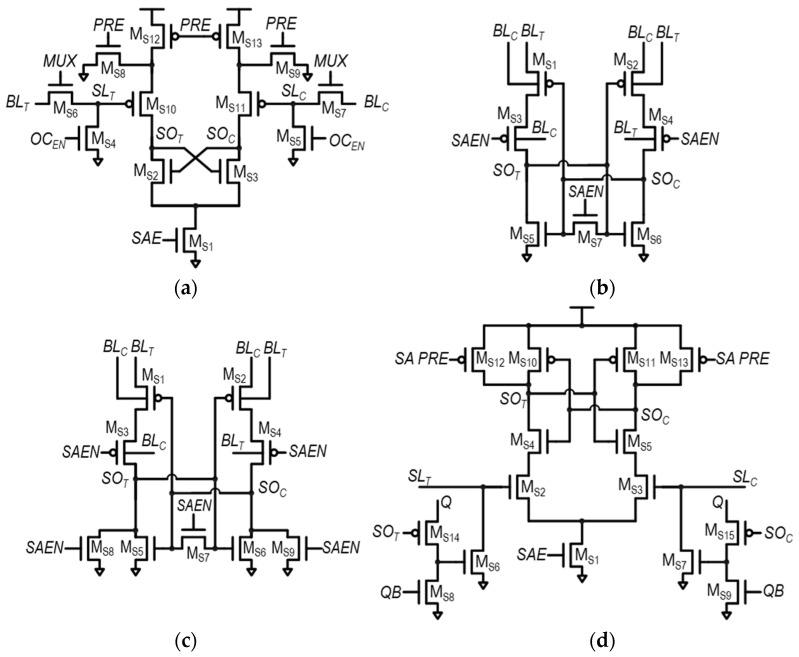
Structure of (**a**) SAOC, (**b**) DIBBSA-FL, (**c**) DIBBSA-PD, and (**d**) CDOR.

In [45], an SA with inherent offset cancellation (SAOC) is proposed, with its structure shown in Figure 17a. The SAOC utilizes pFETS—M_S10_ and M_S11_ in Figure 17a—for input reception, connecting SL_T_ and SL_C_ to the gate node of these pFETs. Before sensing, by driving SL_T_ and SL_C_ low and toggling the PRE from low to high, the |V_thp_| of M_S10_ and M_S11_ is captured at the output nodes of SA—SO_T_ and SO_C_, respectively. Subsequently, BL_T_ and BL_C_ are transferred into SL_T_ and SL_C_ by turned-on MUX transistors, while M_S10_ and M_S11_ are turned on by the low PRE. This results in the charging of SO_T_ and SO_C_ by M_S10_ and M_S11_. In this manner, the SAOC achieves sensing operations, compensating the mismatch between M_S10_ and M_S11_. However, it should be noted that the mismatch between the nFET MUX pair (M_S6_ and M_S7_) is not compensated, and pulling up SL_T_ and SL_C_ with nFETs based on BL_T_ and BL_C_ occurs losses during transmitting BL voltage differences to ΔV_IN,SA_.

In [46], the body-biasing technique is used at critical sensing transistors for auto-offset mitigation features. A differential-input body-biased sense amplifier with floating output nodes (DIBBSA-FL) and a differential-input body-biased sense amplifier with pre-discharge output nodes (DIBBSA-PD) are shown in Figure 17b,c, respectively. The difference between the DIBBSA-FL and the DIBBSA-PD is that the DIBBSA-PD has additional transistors, M_S8_ and M_S9_, to predischarge SO_T_ and SO_C_, while the DIBBSA-FL only equalizes SO_T_ and SO_C_. The operations of DIBBSA-FL and DIBBSA-PD are as follows. During the sensing operation, the SAEB decreases and M_S3_ and M_S4_ turn on. Simultaneously, when BL_T_ is higher than BL_C_, through the body-bias effect on M_S1_, M_S2_, M_S3_, and M_S4_, M_S1_ and M_S3_ become forward body-biased and M_S2_ and M_S4_ become reverse body-biased. Therefore, SO_T_ pulls up much faster than SO_C_. However, recently, 3D FETs such as the FinFET and GAA FET have become commonly used. In these technologies, the body effect is nearly negligible. Therefore, using the body-bias technique in recent technologies is not suitable.

Figure 17d shows the cancellation based on delay and offset relation (CDOR) structure [47]. Before the sensing operation, the mismatch in the SA is captured by the sensing operation, with SL_T_ and SL_C_ equally set to V_DD_. Because of the mismatch in the SA, SO_T_ and SO_C_ become (1, 0) or (0, 1), connected to the gate of M_S15_ and M_S14_, respectively. When SO_T_ and SO_C_ are (1, 0), this means that the pull-up strength on the SO_T_ side is higher than that on the SO_C_ side. Simultaneously, Q and QB become V_DD_ and V_SS_, turning off M_S6_ and M_S7_. In the case of (SO_T_, SO_C_) = (1, 0), M_S14_ turns on and M_S15_ turns off, lowering the SL_T_. Due to the decreased SL_T_, the pull-up strength of the SO_C_ side becomes stronger, which operates as offset mitigation. However, the process of adjusting the voltage is highly challenging. This is because the voltage variance is highly dependent on the offset mitigation activation time and the sizes of the M_S6_ and M_S7_ transistors.

## 4. Comparison

Table 1 summarizes the comparison among the SRAM sensing circuit designs covered in Section 3.

Unlike the conventional SAs (VLSA and CLSA), the STSA, VTSA, and HYSA-QZ drive or pre-charge the internal nodes of the SA in favor of accurate sensing. In this manner, without using additional control signals or employing additional operation phases, the offset voltage can be efficiently reduced. In terms of reducing the *V*_OS_, the VTSA and HYSA-QZ, which directly pre-charge the internal nodes using pass gates connected to SL_T_ and SL_C_, outperform the STSA. This is because the mismatch effects in the gated FETs controlling the SL_T_ and SL_C_ in the STSA are larger than the mismatch effects in the transmission gates used by the VTSA or HYSA-QZ to transfer SL_T_ and SL_C_. Compared with the VTSA, the HYSA-QZ can achieve a smaller *V*_OS_ because more internal nodes are pre-charged than the VTSA. However, the SA delay is increased in the STSA, VTSA, and HYSA-QZ compared to the VLSA, because of using increased stack numbers.

The TMSA, VTS-SA, and CSA_COC_ directly capture mismatches in SAs, utilizing a capacitor(s). In this manner, the *V*_OS_ can be further reduced compared to the STSA, VTSA, and HYSA-QZ. However, this improvement comes at a cost: introducing additional phases or control signals, biasing through short circuit currents, and using large capacitors increase the SA delay and energy consumption significantly. The trade-off between BL delay/energy and SA delay/energy becomes evident in this context. More precise compensation of SA mismatches can result in a smaller *V*_OS_ and reduced BL delay and energy. However, achieving this delicacy requires additional circuit components, which can lead to increased SA delay and energy consumption.

Pre-charging BL circuits, BP^2^SP and CCN-PP, offer an alternative approach to capturing transistor *V*_th_ values and reducing BL voltage development. They can be implemented more simply compared to SA mismatch compensation structures because pre-amplifiers have a simpler structure than SAs. However, controlling BL pre-charge levels can be challenging in practice, especially since they should be floating when diode-connection TRs are used for pre-charging.

In addition to the sensing circuit covered in Section 3, there are several other approaches for reducing V_BL_ requirements [44,45,46,47], as shown in the last four rows in Table 1. However, it is worth noting that these methods have specific characteristics that may affect their applicability. In one of these structures, the SAOC is introduced to address the mismatch between two input pFETs at the beginning of the read access to reduce the *V*_OS_. However, it is important to note that the mismatches other transistor pairs, which are also critical for *V*_OS_, are not able to be compensated. Thus, it may have increased the *V*_OS_ even compared to the conventional SAs. In addition, short-circuit current paths are inevitably formed, which limits its practical applicability.

The OCCSA utilizes the MUX transistors as the common gate amplifier to pre-amplify the V_BL_. Although it is powerful, to operate the MUX as an amplifier, an additional high-voltage source is required for bit-line pre-charge (V_prebl_), which significantly incurs power and area overheads. In addition, to compensate the mismatch between the MUX transistor pair, a significant amount of time is required for the separate bit-line pre-charge phase before the access phase, which substantially degrades the cycle time.

The DIBBSA-FL and DIBBSA-PD are proposed. In these structures, differential bit-line inputs are transferred to differential output nodes through pull-up pFETs, while the body of the output pull-up pFETs are biased with bit-lines to enhance sensing accuracy. However, a critical limitation of these approaches arises from the fact that most recent SRAMs utilize multiple gate FETs, such as finFETs and gate-all-around FETs, which exhibit minimal body effects. Consequently, the current or threshold voltage remains nearly independent of body voltage changes, rendering these structures inapplicable.

The CDOR-based offset compensating sensing circuit is introduced. This structure captures the mismatch in SAs during the pre-charge phase of the SRAM. This is achieved by enabling the SA (SAE = 1) with the condition of *SL*_T_ = *SL*_C_ = *V*_DD_. In this manner, the mismatch information is stored at the differential output nodes, *SO*_T_ and *SO*_C_. For example, if the mismatch favors the SA to make the *SO*_T_ become low, this mismatch capturing process makes *SO*_T_ become 0, while *SO*_C_ becomes high during the pre-charge phase. Then, utilizing this stored information, when the sensing phase starts, *SL*_T_ and *SL*_C_ are calibrated to compensate the mismatch. Although the compensation technique is innovative, the accuracy of this compensation technique is highly dependent on factors such as the width of the calibration timing and the sizing of the calibration transistor. This dependency can potentially result in an increase in the effective *V*_OS_ of the SA, which may render the structure less practical.

**Figure 18:**
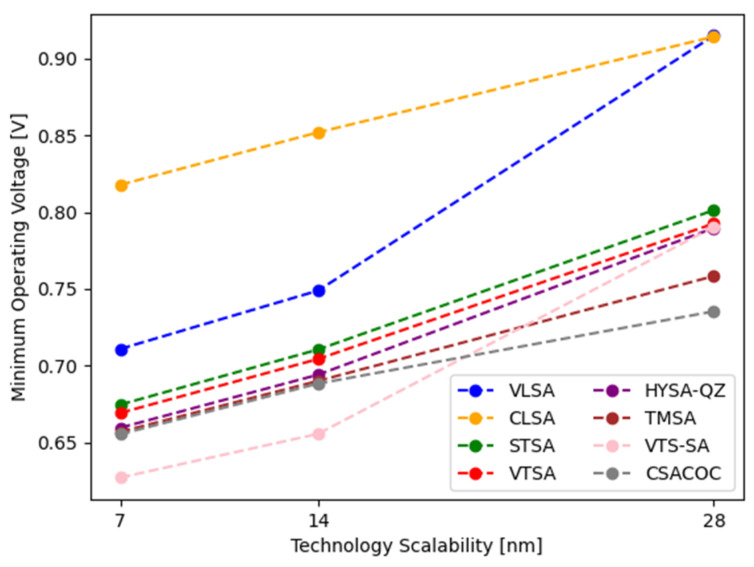
Minimum operating voltage of SAs according to technology scalability.

Figure 18 shows the minimum operating voltage of SAs according to technology scalability. The minimum operating voltage represents the minimum voltage that satisfies the 6σ sensing yield at the operating frequency of 1 GHz in the 7 nm, 14 nm, and 28 nm processes.

A quantitative comparison among the different SAs covered in Section 3 is shown in Table 2. It is simulated in TSMC 28 nm technology when a four-to-one MUX is used, with V_DD_ = 1.0 V, and the number of bit-cells per column is 256. The distribution of *V*_OS_ in the SAs is estimated as follows [63]: First, we assume that *V*_OS_ follows the Gaussian distribution. Thus, P_FailSA_, the probability of sensing failure, can be expressed as follows:

(1) $$P_{\mathrm{FailSA}}=P\left(V_{\mathrm{OS}}{>\;\Delta V}_{\mathrm{IN},\mathrm{SA}}\right)=P\left(\frac{V_{\mathrm{OS}}-\mu_{\mathrm{OS}}}{\sigma_{\mathrm{OS}}}>\frac{{\Delta V}_{\mathrm{IN},\mathrm{SA}}-\mu_{\mathrm{OS}}}{\sigma_{\mathrm{OS}}}\right)=P\left(Z>\frac{{\Delta V}_{\mathrm{IN},\mathrm{SA}}-\mu_{\mathrm{OS}}}{\sigma_{\mathrm{OS}}}\right)$$

in (1), ΔV_IN,SA_ is the SA input voltage difference, *µ*_OS_ is the mean *V*_OS_, *σ*_OS_ is the standard deviation of the *V*_OS_, and *Z* is the standard Gaussian random variable. Second, representing the standard Gaussian cumulative distribution function (CDF) as Φ(z), Equation (1) can be shown as follows:

(2) $$P_{\mathrm{FailSA}}=1-\Phi \left(\frac{{\Delta V}_{\mathrm{IN},\mathrm{SA}}-\mu_{\mathrm{OS}}}{\sigma_{\mathrm{OS}}}\right)$$

Third, through the inverse function, (2) can be expressed as follows:

(3) $$\mu_{\mathrm{OS}}+\sigma_{\mathrm{OS}}\Phi^{-1}\left(1-P_{\mathrm{FailSA}}\right)=\Delta V_{\mathrm{IN},\mathrm{SA}}$$

in (3), both *P*_failSA_ and ΔV_IN,SA_ are values obtainable through simulation. With the specified values for *P*_failSA_ and ΔV_IN,SA_, only *µ*_OS_ and *σ*_OS_ remain as variables in (3). Thus, with two instances of (3), the two variables, *µ*_OS_ and *σ*_OS_, can be derived. Therefore, due to a 1000-sample Monte Carlo simulation of V_INtest1_ (ΔV_IN,SA_ = 10 mV) and V_INtest2_ (ΔV_IN,SA_ = −10 mV), *P*_FailSA1_ and *P*_FailSA2_ can be determined and can be shown as the following two equations, using (3):

(4) $$\mu_{\mathrm{OS}}+\sigma_{\mathrm{OS}}\Phi^{-1}\left(1-P_{\mathrm{FailSA}}{}_{1}\right)=V_{\mathrm{INtest}}{}_{1}$$

(5) $$\mu_{\mathrm{OS}}+\sigma_{\mathrm{OS}}\Phi^{-1}\left(1-P_{\mathrm{FailSA}}{}_{2}\right)=V_{\mathrm{INtest}}{}_{2}$$

Finally, by calculating (4) and (5), *μ*_OS_ and *σ*_OS_ can be shown as follows:

(6) $$\mu_{\mathrm{OS}}=\frac{\Phi^{-1}\left(1-P_{\mathrm{FailSA}}{}_{2}\right)V_{\mathrm{INtest}}{}_{1}-\Phi^{-1}\left(1-P_{\mathrm{FailSA}}{}_{1}\right)V_{\mathrm{INtest}}{}_{2}}{\Phi^{-1}\left(1-P_{\mathrm{FailSA}}{}_{2}\right)-\Phi^{-1}\left(1-P_{\mathrm{FailSA}}{}_{1}\right)}$$

(7) $$\sigma_{\mathrm{OS}}=\frac{V_{\mathrm{INtest}}{}_{1}-V_{\mathrm{INtest}}{}_{2}}{\Phi^{-1}\left(1-P_{\mathrm{FailSA}}{}_{1}\right)-\Phi^{-1}\left(1-P_{\mathrm{FailSA}2}\right)}$$

In (6) and (7), because V_INtest1_, V_INtest2_, *P*_FailSA1_, and *P*_FailSA2_ are determined through simulation, *μ*_OS_ and *σ*_OS_ can be estimated. Additionally, the energy consumption is measured by integrating the sum of all currents flowing during one cycle. The energy consumptions shown correspond to those consumed at the four columns of the BL. As mentioned earlier, the reduction in *V*_OS_ can be observed to enhance the performance of BL delay/energy and SA delay/energy.

## Figures and Tables

**Table 1:** Comparison of SRAM sensing circuit designs.

	Structure	Offset Reduction Technique	Components	Control Signals	Limitations
VLSA	Figure 2a	-	7 TR	*PCB*, *SAE*	Large *V*_OS_
CLSA	Figure 2b	-	9 TR	*PCB*, *SAE*	Increased *V*_OS_ due to additional TR pair
STSA	Figure 6a	Driving Internal Nodes of VLSA (Z_T_ and Z_C_)	11 TR	*PCB*, *SAE*	Speed degradation due to stack
VBSTSA	Figure 6b	STSA + Negative Boosting *V*_SS_	14 TR + NVG (share)	*PCB*, *SAE*, *BSTEN*	Necessitating for NVG (power/area cost)
VTSA	Figure 7a	Pre-charging *SO*_T_ and *SO*_C_ to *SL*_T_ and *SL*_C_ in CLSA	9 TR	*PCB*, *SAE*	Speed degradation due to stack
HYSA-QZ	Figure 7b	Pre-charging output nodes and internal nodes of CLSA	11 TR	*PCB*, *SAE*	Speed degradation due to stack
TMSA	Figure 8a	Capturing *V*_th_ of pull-down nFETs through paired cap	11 TR + INV + Buffer + 2 C	*PCB*, *SAE*	Capacitor mismatch, Cap power/area overhead
VTS-SA	Figure 10	Capturing trip points of cross-coupled INVs with input acceptation via coupling cap pair	12 TR + 2 C	*EN*, *PCB*, *PRE*, *SAE*	Capacitor mismatch, power/area overhead
CSA_COC_	Figure 12a	Capturing trip points of cross-coupled inverters via single capacitor	16 TR + 1C +2 OR (shared)	*PCB*, *SAE*, *Φ*_Trs_, *Φ*_Trb_	Many switches, control signal circuit
BP^2^SP	Figure 14a	Capturing V_th_ of pre-amplifying pFET pair at BL pre-charge	6TR + SA	*PCB*, *SAE*	Bit-line floating, unstable pre-charge level, power/area overhead
CCN-PP	Figure 15a	Pre-amplifying BL via cross-coupled nFET pair, while capturing V_th_ with boosted *V*_DD_	4TR + 2C + Buffer + 1TR + SA	*PCB*, *SAE*, *PBE*	Bit-line floating, power/area overhead
OCCSA	[44]	Capturing *V*_th_ of MUX nFETs at BL pre-charge	7 TR	*PCB*, *SAE*	Additional Vprebl voltage generator, different MUX signal
SAOC	[45]	Capturing *V*_th_ of input pFETs at SA pre-charge	11 TR	*PCB*, *SAE*, *OCEN*	N1, N2 mismatch, control signal circuit
DIBBSA-FL, DIBBSA-PD	[46]	Body biasing	7 TR, 9TR +Body contact	*PCB*, *SAE*	Inapplicable to the recent technology whose body effect is minimal
CDOR	[47]	Lowering input voltage according to SA mismatch	15 TR	*PCB*, *SAE*, *Q*	Control signal circuit for added Q and different PCB, SAE operation

**Table 2:** Quantitative comparison of SRAM SAs at V_DD_ = 1.0 V in 28 nm technology.

	Standard Dev. of *V*_OS_ (mV)	BL Delay (ps)	SA Delay (ps)	Energy Consumption for Four BLs (fJ)	SA Energy Consumption (fJ)	Area (µm^2^)
VLSA	16.46	203.86	15.25	93.86	2.94	6.48
CLSA	27.77	323.32	27.69	110.35	3.99	7.88
STSA	12.24	159.57	19.41	86.90	3.67	8.49
VTSA	11.54	152.21	17.67	87.47	3.37	6.79
HYSA-QZ	10.39	140.25	16.83	79.21	3.65	7.09
TMSA	9.96	138.46	13.47	95.43	25.23	8.63
VTS-SA	5.75	91.84	15.25	76.60	16.38	7.11
CSA_COC_	9.76	133.67	27.69	89.27	18.26	9.55

## Data Availability

Not applicable.
